# Sporadic multiple meningiomas harbor distinct driver mutations

**DOI:** 10.1186/s40478-020-01113-2

**Published:** 2021-01-06

**Authors:** Tareq A. Juratli, Insa Prilop, Felix C. Saalfeld, Sylvia Herold, Matthias Meinhardt, Carina Wenzel, Silke Zeugner, Daniela E. Aust, Fred G. Barker, Daniel P. Cahill, Priscilla K. Brastianos, Sandro Santagata, Gabriele Schackert, Thomas Pinzer

**Affiliations:** 1 Department of Neurosurgery, University Hospital Carl Gustav Carus, Technische Universität Dresden, Fetscherstr. 74, 01307 Dresden, Germany; 2 Translational Neuro-Oncology Laboratory, Department of Neurosurgery, Massachusetts General Hospital, Harvard Medical School, Boston, MA USA; 3 Clinic of Internal Medicine I, Dresden University Hospital, Dresden, Germany; 4 Core Unit for Molecular Tumor Diagnostics (CMTD), National Center for Tumor Diseases Dresden (NCT/UCC), Dresden, Germany; 5 Biobank Dresden, National Center for Tumor Diseases Dresden (NCT/UCC), Dresden, Germany; 6 Department for Pathology, Dresden University Hospital, Dresden, Germany; 7 Department of Neurosurgery, Massachusetts General Hospital, Harvard Medical School, Boston, MA USA; 8 Division of Hematology/Oncology, Department of Neurology, Stephen E. and Catherine Pappas Center for Neuro-Oncology, Massachusetts General Hospital, Harvard Medical School, Boston, MA USA; 9 Department of Pathology, Brigham and Women’s Hospital, Boston, MA USA

In a small fraction of patients, intracranial meningiomas arise as multiple and spatially distinct masses therefore presenting a unique management challenge [9, 12, 19]. A recently-published, (Surveillance, Epidemiology, and End Results) SEER-based study has reported that patients with multiple meningiomas (MM) have substantially reduced overall survival when compared to patients with single meningiomas [14]. Patients may develop multiple meningiomas in sporadic or hereditary forms. Familial syndromes that are commonly associated with MM are neurofibromatosis type 2 (NF2) and familial meningiomatosis in patients with germline *NF2* and *SMARCB1* mutations, respectively [2, 15]. While the mutational landscape of single meningiomas has been extensively studied [3–5, 10, 20], understanding of the molecular pathogenesis of sporadic MM remains incomplete. Older studies and case reports have reported molecular testing in patients with sporadic MM that have principally been focused on tumors with *NF2* mutations [8, 16–18]. However, to our knowledge, no molecular profiling in a case series of spatially separated MM, composed of different histological subtypes, has been performed. The objective of this study is to elucidate the genetic features of sporadic MM, defined as the presence of ≥ 2 spatially separated synchronous or metachronous lesions.

This series includes 17 resected sporadic meningiomas from eight patients (seven females and one male) that were identified by a record search for patients with MM. All patients presented with synchronous, spatially separated meningiomas without evidence of tumor bridging, as reviewed on MR-imaging. The patients had no significant prior radiation exposure and the tumors did not arise in patients who met the clinical criteria for the diagnosis of familial schwannomatosis or neurofibromatosis type 2 [6]. In addition, upon reviewing cranial and spinal MR images, no patient had other intra- or extra-cranial tumors associated with hereditary meningioma syndromes such as schwannomas or ependymomas.

Fresh frozen tumor tissue was available from all 17 meningiomas and was retrieved from the archives of the Institute for Pathology at the University Hospital Dresden upon approval of the local ethics committee. Two board-certified pathologists confirmed the pathologic diagnosis of each case. All tumors were classified according to the 2016 WHO classification of tumors of the central nervous system [11]. The tumor DNA was purified using AllPrep^®^ DNA Universal Kit for fresh frozen tissue (Qiagen, Germantown MD) following the manufacturer’s instructions. The regions of interest were amplified using a custom designed amplikon panel according to the protocol “QIAseq Targeted DNA V3 Panel, May 2017” (QIAGEN, Hilden, Germany). The panel was custom-designed by our group and manufactured by QIAGEN. The panel covers either mutation hotspots or—where loss of function is a known mechanism of action—whole genes. The following meningioma-relevant genes are included: *AKT1, ATRX, CDKN2A, KLF4, NF1, NF2, PIK3CA, PIK3R1, POLR2A, PTEN, SMARCB1, SMO, STAG2, SUFU, TP53, TRAF7*, and *TERT* promotor. During library preparation unique molecular barcodes and sample specific indices were incorporated according to the protocol. Indexed libraries were then quantified using a Qubit dsDNA HS Assay Kit (Thermo Fisher Scientific, MA, USA) and paired end sequenced (2x200 bp) on Illumina MiSeq platform. HG19 was used as reference genome for bioinformatic analyses. The bioinformatics evaluation was performed using the Biomedical Workbench from CLC (12.0.3) using a customized analysis algorithm with following filters: coverage >/=100, allele frequency >/=5%. Notably, we performed internal NGS controls for identity check and cross contamination checks to assure the assignment of the correct samples.

The average age at presentation was 60 years (range 43–75 years) which is comparable with the age of patients with single sporadic meningiomas [9]. Six patients (75%) underwent two surgeries within 2 years for tumor resection, whereas in two patients the meningiomas were removed at the same time (patients 1 and 7). Fourteen meningiomas were WHO grade 1 (82.3%) and the remaining three tumors were WHO grade 2. This is consistent with previous reports of the predominance of WHO grade 1 among MM [13, 19].

**Fig. 1:**
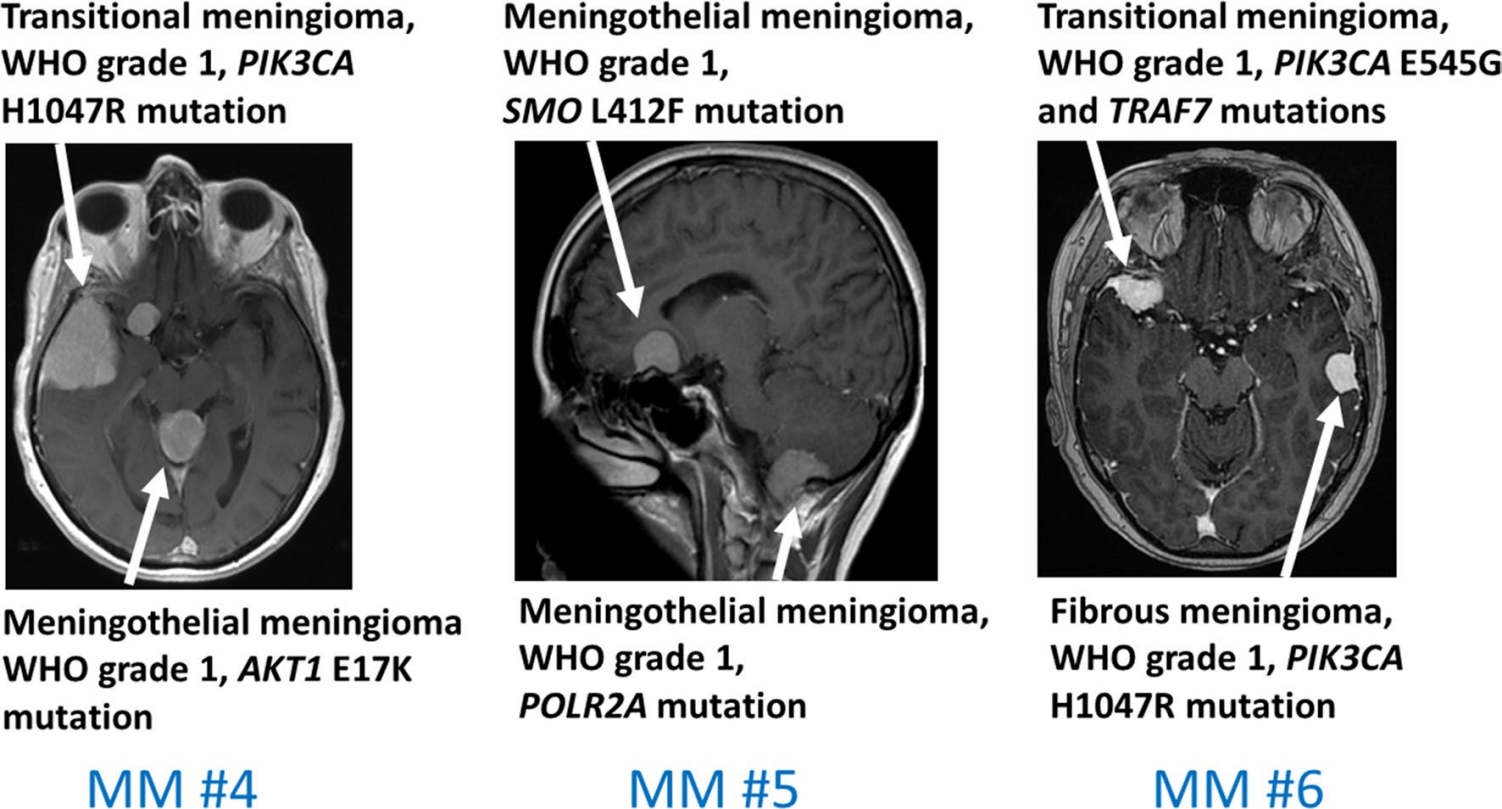
Illustrative cases from three patients with seven meningiomas are shown. No separate tumors within individual patients shared driver mutations

Most importantly, the same mutation was not identified in separate tumors from the same patient, suggesting genomically distinct molecular drivers and an independent origin of these multiple lesions. All but two cases harbored *TRAF7*, *AKT1*, *SMO* or *PIK3CA* mutations (Fig. 1). The most frequent driver mutations in our series were *TRAF7* (n = 5), *PIK3CA* H1047R and E545G (n = 3), *AKT1* E17K (n = 3), *NF2* (n = 2), *SMO* L412F (one case) and *NF1* (one case). We did not detect a known driver mutation in only one meningioma (MM #3, Site B; Table 1). Interestingly, with the exception of one patient (MM #5), all tumors from the same patient were different histopathological subtypes (Table 1).

**Table 1:** Patients’ and tumor characteristics

Patient	Age (years)	Sex	Tumor site	Tumor location	WHO grade	Histologic features	Mutations and allele frequency (%)
MM#1	71	F	Site-A	Infratentorial (L)	1	Transitional	*AKT1* p.E17K (33%)
			Site-B	Petroclival (L)	1	Meningothelial	*TRAF7* p.K615N (29%)
MM#2	43	F	Site-A	Convexity (R)	1	Microcystic	*NF1* p.F384L (5%)
			Site-B	Anterior SB (L)	1	Meningothelial/transitional	*NF2* p.A325fs (37%)
MM#3	62	M	Site-A	Convexity (R)	2	Atypical	*NF2* p.E541* (32%)
			Site-B	Petrosal (R)	1	Fibrous	None
MM#4	70	F	Site-A	Sphenoid wing (R)	1	Transitional	*PIK3CA* p.H1047R (36%)
			Site-B	Pineal region (M)	1	Meningothelial	*AKT1* p.E17K (36%)
MM#5	49	F	Site-A	Foramen magnum (M)	1	Meningothelial	*POLR2A* p.H439del (26%)
			Site-B	Olfactory groove	1	Meningothelial	*SMO* p.L412F (40%)
MM#6	53	F	Site-A	Sphenoid wing (R)	1	Transitional	*PIK3CA* p.H1047R (35%), *TRAF7* p.S537F (38%)
			Site-B	Planum spenoidale (M)	1	Mixed fibrous/meningothelial	*PIK3CA* p.E545G (30%)
			Site-C	Convexity (L)	1	Fibrous	*PIK3CA* p.H1047R (38%)
MM#7	75	F	Site-A	Petroclival (L)	2	Chordoid	*AKT1* p.E17K (31%)
			Site-B	Foramen magnum (M)	2	Transitional	*TRAF7* p.R641H (33%)
MM#8	54	F	Site-A	Convexity (L)	1	Meningothelial	*TRAF7* p.Q38E (35%)
			Site-B	Convexity (R)	1	Secretory	*TRAF7* p.N520S (41%), *KLF4* p.K409Q (40%)

*R* right, *L* left, *M* midline

The low frequency of *NF2* mutations in our MM series stands in contrast to previous studies that included hereditary cases arising in the setting of NF2 [2, 15, 16, 18]. Those studies identified a high prevalence of *NF2* mutations (up to 83%) and supported a monoclonal origin for MM [8, 18]. Our findings in a cohort of 17 MM arising in patients without NF2 support a model in which sporadic MM can arise independently from one another, while a subset of MM may result from somatic *NF2* mosaicism [7].

Each of the meningiomas in our study exhibited features that are commonly seen in solitary meningiomas, demonstrating strong associations between the genetic alteration, the histologic subtype and the anatomic location [1, 5, 21]. The high frequency of known and targetable drivers of meningioma in our cohort suggests that a large fraction of MM may be candidates for study in clinical trials evaluating targeted therapies, such as the ongoing multicenter phase II study (ClinicalTrials.gov NCT02523014) that investigates the efficacy of afuresertib in *AKT1*-mutant, vismodegib in *SMO*-mutant and the focal adhesion kinase (FAK) inhibitor GSK2256098 in *NF2*-mutant meningiomas. Given the inter-tumor and intra-patient heterogeneity that we observe in the setting of MM, target lesions should be genomically characterized and not assumed to share molecular alterations with separately resected lesions.

Taken together, our molecular analysis supports the genomic divergence of sporadic MM and presumably their independent origin. Our findings have important clinical implications for this patient population and suggests molecular stratification of each meningioma lesion in patients with sporadic MM to improve the design of meningioma clinical trials and help improve patient management.
